# K_Ca_3.1 upregulation preserves endothelium‐dependent vasorelaxation during aging and oxidative stress

**DOI:** 10.1111/acel.12502

**Published:** 2016-06-30

**Authors:** Shinkyu Choi, Ji Aee Kim, Hai‐yan Li, Kyong‐Oh Shin, Goo Taeg Oh, Yong‐Moon Lee, Seikwan Oh, Yael Pewzner‐Jung, Anthony H. Futerman, Suk Hyo Suh

**Affiliations:** ^1^ Department of PhysiologyMedical SchoolEwha Womans UniversitySeoulSouth Korea; ^2^ College of Pharmacy and MRCChungbuk National UniversityChongjuSouth Korea; ^3^ Department of Life SciencesEwha Womans UniversitySeoulSouth Korea; ^4^ Department of Molecular MedicineMedical SchoolEwha Womans UniversitySeoulSouth Korea; ^5^ Department of Biological ChemistryWeizmann Institute of ScienceRehovotIsrael

**Keywords:** aging, Ca^2+^‐activated K^+^ channel, ceramide synthase 2 ablation, endothelial cells, oxidative stress, redox enzymes

## Abstract

Endothelial oxidative stress develops with aging and reactive oxygen species impair endothelium‐dependent relaxation (EDR) by decreasing nitric oxide (NO) availability. Endothelial K_Ca_3.1, which contributes to EDR, is upregulated by H_2_O_2_. We investigated whether K_Ca_3.1 upregulation compensates for diminished EDR to NO during aging‐related oxidative stress. Previous studies identified that the levels of ceramide synthase 5 (CerS5), sphingosine, and sphingosine 1‐phosphate were increased in aged wild‐type and CerS2 mice. In primary mouse aortic endothelial cells (MAECs) from aged wild‐type and CerS2 null mice, superoxide dismutase (SOD) was upregulated, and catalase and glutathione peroxidase 1 (GPX1) were downregulated, when compared to MAECs from young and age‐matched wild‐type mice. Increased H_2_O_2_ levels induced Fyn and extracellular signal‐regulated kinases (ERKs) phosphorylation and K_Ca_3.1 upregulation. Catalase/GPX1 double knockout (catalase^−/−^/GPX1^−/−^) upregulated K_Ca_3.1 in MAECs. NO production was decreased in aged wild‐type, CerS2 null, and catalase^−/−^/GPX1^−/−^ MAECs. However, K_Ca_3.1 activation‐induced, *N*^*G*^‐nitro‐l‐arginine‐, and indomethacin‐resistant EDR was increased without a change in acetylcholine‐induced EDR in aortic rings from aged wild‐type, CerS2 null, and catalase^−/−^/GPX1^−/−^ mice. CerS5 transfection or exogenous application of sphingosine or sphingosine 1‐phosphate induced similar changes in levels of the antioxidant enzymes and upregulated K_Ca_3.1. Our findings suggest that, during aging‐related oxidative stress, SOD upregulation and downregulation of catalase and GPX1, which occur upon altering the sphingolipid composition or acyl chain length, generate H_2_O_2_ and thereby upregulate K_Ca_3.1 expression and function via a H_2_O_2_/Fyn‐mediated pathway. Altogether, enhanced K_Ca_3.1 activity may compensate for decreased NO signaling during vascular aging.

## Introduction

Endothelial oxidative stress develops with aging and thereby impairs endothelial function (Donato *et al*., 2007; Ungvari *et al*., 2010). Endothelial cells (ECs) contribute to the maintenance of vascular homeostasis by secreting nitric oxide (NO), prostacyclin, and endothelium‐derived hyperpolarizing factor, thereby playing an important role in preventing the genesis and progression of cardiovascular diseases. Although various factors have been shown to cause endothelial dysfunction, some evidence supports the role of reactive oxygen species (ROS) or oxidative stress in the dysfunction (Gori & Munzel, 2011). Dysregulated redox signaling and increased ROS production with aging lead to endothelial dysfunction, thereby contributing to the pathogenesis of cardiovascular diseases, such as coronary artery diseases, hypertension, and atherosclerosis, in elderly patients (Ungvari *et al*., 2010). An increase in ROS generation reduces NO bioavailability through several mechanisms including a direct interaction between NO and ROS, resulting in vascular dysfunction (Donato *et al*., 2007; Ungvari *et al*., 2008; Seals *et al*., 2011). However, little is known about the mechanism by which ECs preserve their function of relaxing vascular smooth muscle during aging‐related oxidative stress.

Reactive oxygen species, generated in response to various stimuli, are scavenged by the endogenous antioxidant enzymes, such as superoxide dismutase (SOD), catalase, and glutathione peroxidase (GPX). Antioxidant enzymes are present in lipid rafts on cell membranes or have close relationships with lipid rafts (Li & Zhang, 2013). In addition, redox molecules, such as the NADPH oxidase subunits or cofactors, are present in lipid rafts (Li & Zhang, 2013). This evidence supports the view that lipid rafts are involved in initiating and transmitting redox signaling in cells. Recent studies have demonstrated that altering the sphingolipid composition by ceramide synthase 2 (CerS2) ablation affects lipid rafts (Park *et al*., 2013) and results in ROS generation through the modulation of mitochondrial complex IV activity (Zigdon *et al*., 2013). In addition, sphingolipid composition and the activities of antioxidant enzymes (SOD, catalase, GPX) were altered in aged rats or rabbits (Lightle *et al*., 2000; Cejkova *et al*., 2004). Furthermore, our previous study showed that the levels of ceramide synthases (CerSs) and sphingolipid composition are altered with aging in mice and that aging‐related alteration in the levels of CerSs and sphingolipid composition is similar to the alteration induced by CerS2 ablation (Choi *et al*., 2015b). These results suggest that ROS (which are generated as a result of altered sphingolipid composition) play an important role in the aging process, and that CerS2 null mice can allow us to investigate aging‐related and altered sphingolipid composition‐induced changes in cellular functions.

Ca^2+^‐activated K^+^ channels (K_Ca_2.3 and K_Ca_3.1) affect endothelial function by modulating endothelium‐dependent responses including endothelium‐dependent hyperpolarization and NO release (Busse *et al*., 2002; Climent *et al*., 2014). Endothelial hyperpolarization induced by K^+^ channel activation or endothelium‐derived hyperpolarizing factor hyperpolarizes vascular smooth muscle cells to induce endothelium‐dependent relaxation (EDR). In addition, endothelial hyperpolarization may promote Ca^2+^ influx through Ca^2+^ entry channels in ECs (Behringer & Segal, 2015), thereby increasing NO production (Sheng *et al*., 2009). Thus, the impairment of K_Ca_2.3 and K_Ca_3.1 affects the endothelium‐dependent control of vascular contractility, which results in a predisposition to vascular diseases (Grgic *et al*., 2009). An increase in blood pressure has been reported in K_Ca_3.1 knockout mice (Si *et al*., 2006), and we have previously suggested that K_Ca_3.1 downregulation is a cause of endothelial dysfunction in Fabry disease (Park *et al*., 2011). In addition, we reported that superoxide generated from ECs downregulates K_Ca_3.1, resulting in endothelial dysfunction in preeclampsia (Choi *et al*., 2013a). In contrast, H_2_O_2_ upregulates K_Ca_3.1 via an ERK‐mediated pathway (Choi *et al*., 2013a,b). Thus, ROS affect endothelial function by modulating endothelial K_Ca_3.1 expression. As ROS generation is increased with aging (Ungvari *et al*., 2010), aging‐related ROS production might affect endothelial function by modulating endothelial K_Ca_3.1 expression. However, signaling pathways that modulate endothelial K_Ca_3.1 expression and function during conditions of age‐related oxidative stress remain undefined.

This study focused on the effect of H_2_O_2_ on the endothelial function of inducing the relaxation of vascular smooth muscle using mouse aortic endothelial cells (MAECs) from aged wild‐type and CerS2 null mice, in which sphingolipid composition is altered and ROS production is increased. The results showed that a H_2_O_2_‐induced increase in K_Ca_3.1 activity compensated for diminished EDR to NO under aging‐related oxidative stress conditions.

## Results

### ROS generation in MAECs from aged wild‐type and young Cer2 null mice

**Figure 1:**
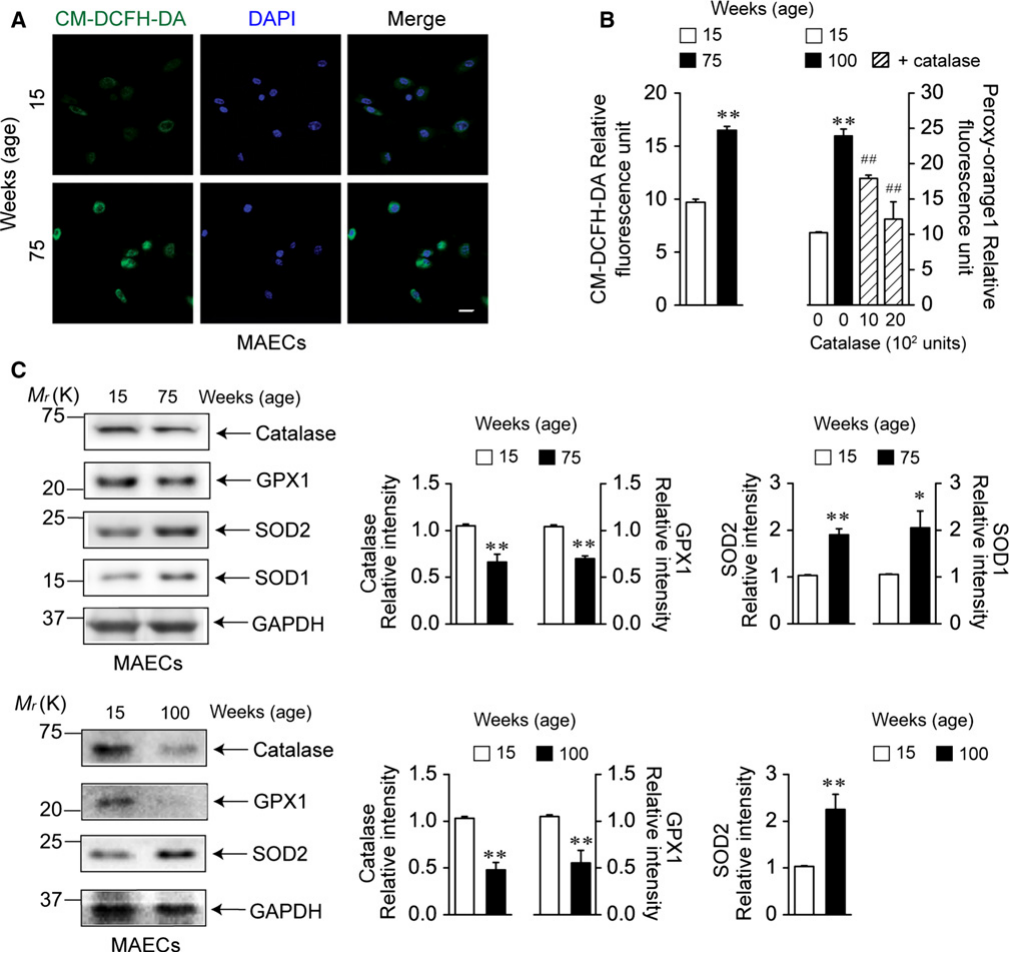
Levels of reactive oxygen species and antioxidant enzymes in aged wild‐type mouse aortic endothelial cells (MAECs). H_2_O_2_ production and levels of antioxidant enzymes were examined in MAECs from young (15‐week‐old) and aged (75‐ and 100‐week‐old) wild‐type mice. (A) Fluorescence was detected by confocal laser microscopy. Nuclei were stained with DAPI (blue). Compared with young wild‐type MAECs, the green fluorescence indicative of H_2_O_2_ was markedly increased in aged wild‐type MAECs. Scale bar: 20 μm. (B) Fluorescence was detected by a microplate fluorescence reader. Results were observed in each set of five different cultures. (C) Protein levels of catalase, GPX1, SOD1, and SOD2 were measured. Blots are representatives of three to four experiments performed with three to four different cultures. Results were normalized to GAPDH levels. *P < 0.05, **P < 0.01 vs. young wild‐type MAECs, ^##^ *P *< 0.01 vs. 100‐week‐old wild‐type MAECs.

**Figure 2:**
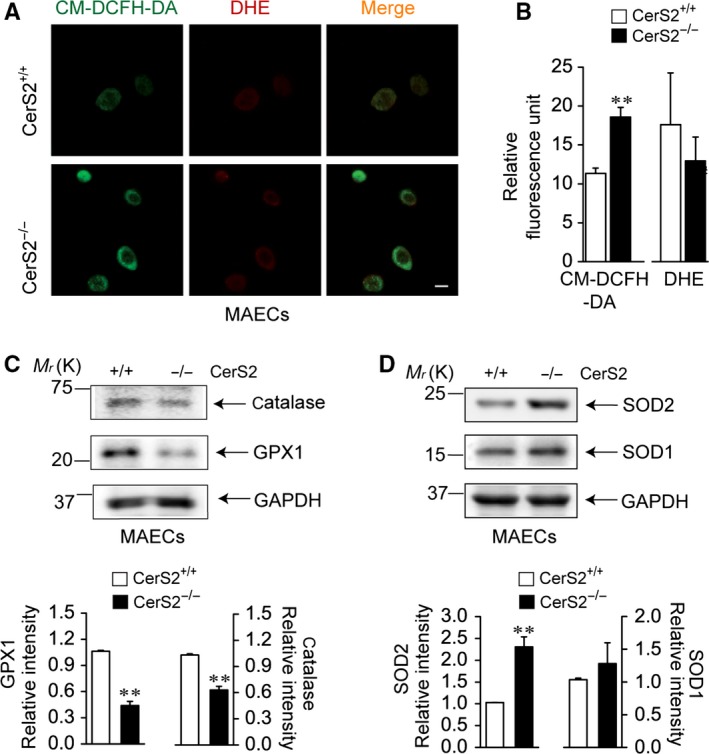
Changes in the levels of reactive oxygen species (ROS) and antioxidant enzymes caused by CerS2 ablation. Primary cultured mouse aortic endothelial cells (MAECs) isolated from young (25‐week‐old) CerS2 null and age‐matched wild‐type mice were used to examine ROS generation (A,B) and levels of antioxidant enzymes (C,D). (A) Fluorescence was detected by confocal laser microscopy. The green fluorescence indicative of H_2_O_2_ was significantly increased, but the red fluorescence indicative of superoxide was not when compared to wild‐type MAECs. Scale bar: 10 μm. (B) Fluorescence was detected by a microplate fluorescence reader. Results were observed in each set of five different cultures. (C,D) Protein levels of catalase, GPX1 (C), SOD1, and SOD2 (D) were examined. Blots are representatives of three experiments performed with three different cultures, and the results were normalized to GAPDH levels. **P < 0.01 vs. wild‐type MAECs. GPX1, glutathione peroxidase 1; SOD, superoxide dismutase.

We investigated whether ROS generation is increased in MAECs from aged wild‐type (Fig. 1) and young CerS2 null (Fig. 2) mice using the H_2_O_2_‐sensitive dye, 5‐(and 6‐) chloromethyl‐2′,7′‐dichlorofluorescin diacetate (CM‐DCFH‐DA) or peroxy‐orange 1, or the superoxide‐sensitive dye, dihydroethidine. Compared with young wild‐type MAECs, H_2_O_2_ levels were markedly increased in aged wild‐type MAECs (Fig. 1A,B). The increased H_2_O_2_ levels were reduced by treatment with catalase (Fig. 1B). In aged wild‐type MAECs, catalase and GPX1 levels were decreased, whereas levels of SOD1 and SOD2 were significantly increased (Fig. 1C). A previous study reported that thioredoxin 1 protein levels are increased in aged ECs (Altschmied & Haendeler, 2009). In young CerS2 null MAECs, H_2_O_2_ levels were significantly increased, but superoxide levels were not when compared to age‐matched wild‐type MAECs (Fig. 2A,B). We then examined whether levels of antioxidant enzymes are altered in CerS2 null MAECs. In young CerS2 null MAECs, catalase and GPX1 were downregulated (Fig. 2C), whereas the mitochondrial SOD, SOD2, was upregulated (Fig. 2D). In addition, mRNA levels of thioredoxin 1 and thioredoxin 2 were significantly decreased (Fig. S1). On the other hand, levels of the cytoplasmic SOD, SOD1, were unchanged. As SOD upregulation might increase degradation of superoxide to H_2_O_2_, and downregulation of catalase, GPX1, and thioredoxins is indicative of decreased degradation of H_2_O_2_, these results suggest that H_2_O_2_ levels are increased in aged wild‐type and young CerS2 null MAECs.

### H_2_O_2_, generated by alteration in the levels of antioxidant enzymes, upregulates K_Ca_3.1

**Figure 3:**
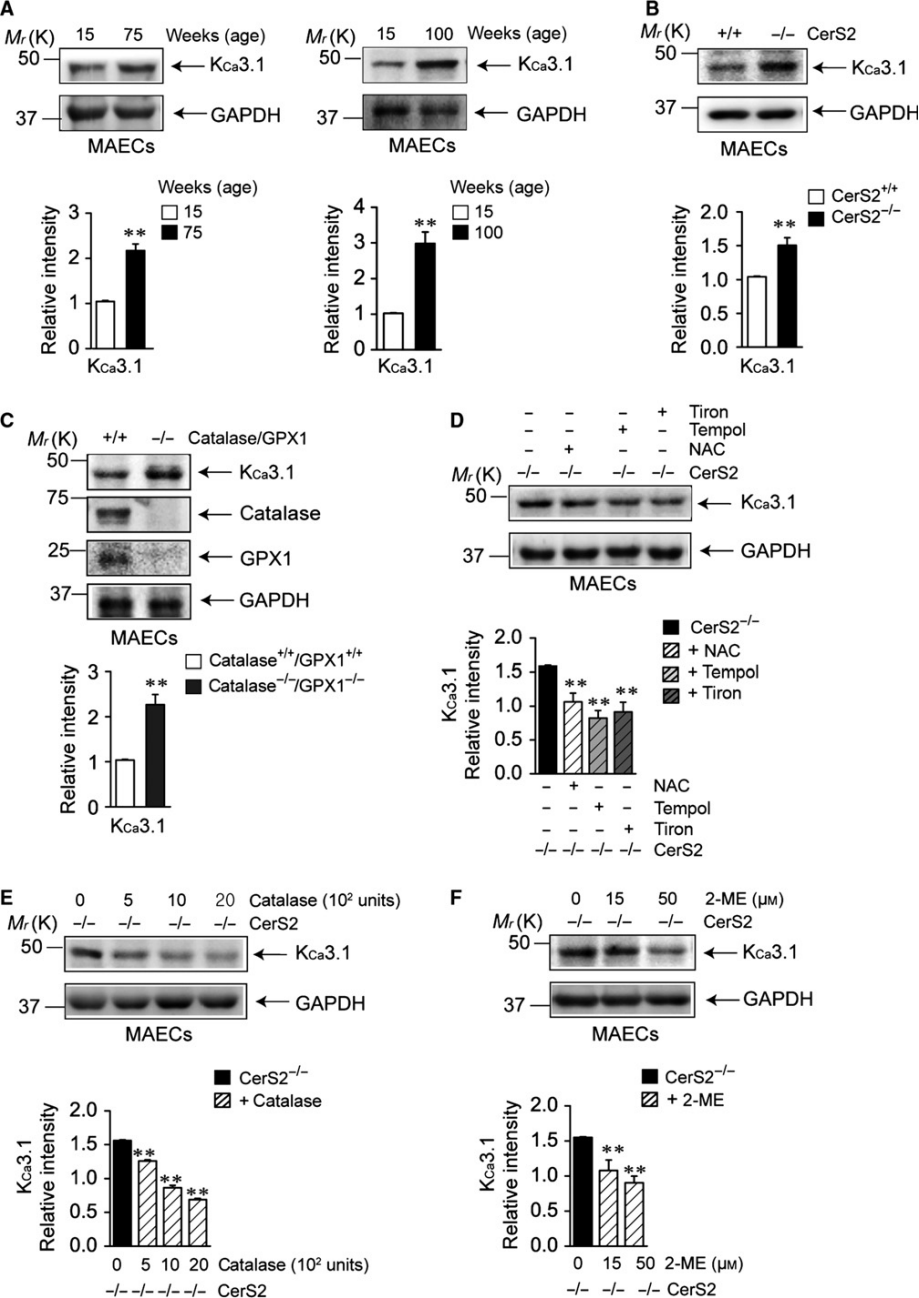
H_2_O_2_ upregulates endothelial K_C__a_3.1. Protein levels of K_C__a_3.1 were examined in mouse aortic endothelial cells (MAECs) from young and aged wild‐type mice (A), in MAECs from young CerS2 null and age‐matched wild‐type mice (B), in MAECs from young catalase^−/−^/GPX1^−/−^ and age‐matched wild‐type mice (C), in CerS2 null MAECs treated with tiron, tempol, or NAC (D), or in CerS2 null MAECs treated with catalase (E) or 2‐ME (F). Blots are representatives of four experiments performed with four different cultures. Results were normalized to GAPDH levels. **P < 0.01 vs. young, age‐matched wild‐type (A–C), or vehicle‐treated CerS2 null (D–F) MAECs.

In our previous studies, we reported that K_Ca_3.1 is upregulated by H_2_O_2_ and downregulated by superoxide (Choi *et al*., 2013a,b), suggesting the possibility of endothelial K_Ca_3.1 upregulation by H_2_O_2_ in aged wild‐type and young CerS2 null MAECs. Thus, we examined K_Ca_3.1 levels in aged wild‐type and young CerS2 null MAECs. K_Ca_3.1 expression was significantly upregulated in aged wild‐type (Fig. 3A) and CerS2 null (Fig. 3B) MAECs. As catalase and GPX1 were downregulated in aged wild‐type and CerS2 null MAECs, we examined whether knockouts of catalase and GPX1 affect endothelial K_Ca_3.1 levels using young catalase/GPX1 double‐knockout (catalase^−/−^/GPX1^−/−^) mice. K_Ca_3.1 was markedly upregulated in catalase^−/−^/GPX1^−/−^ MAECs (Fig. 3C), indicating that downregulation of catalase and GPX1 plays a critical role in K_Ca_3.1 upregulation in aged wild‐type and young CerS2 null mice. In addition, K_Ca_3.1 upregulation in CerS2 null MAECs was reduced by inhibition of ROS generation. Treatment with tiron, tempol, or *N*‐acetyl‐cysteine (NAC) reduced K_Ca_3.1 levels (Fig. 3D). Furthermore, treatment with catalase (Fig. 3E) or 2‐methyl estradiol (2‐ME) (Fig. 3F) reduced K_Ca_3.1 levels in a concentration‐dependent manner. These results suggest that changing the levels of antioxidant enzymes (SOD2 upregulation and downregulation of catalase and GPX1) increases H_2_O_2_ levels and thereby K_Ca_3.1 levels.

### Fyn and ERK activation by H_2_O_2_ induces K_Ca_3.1 upregulation

**Figure 4:**
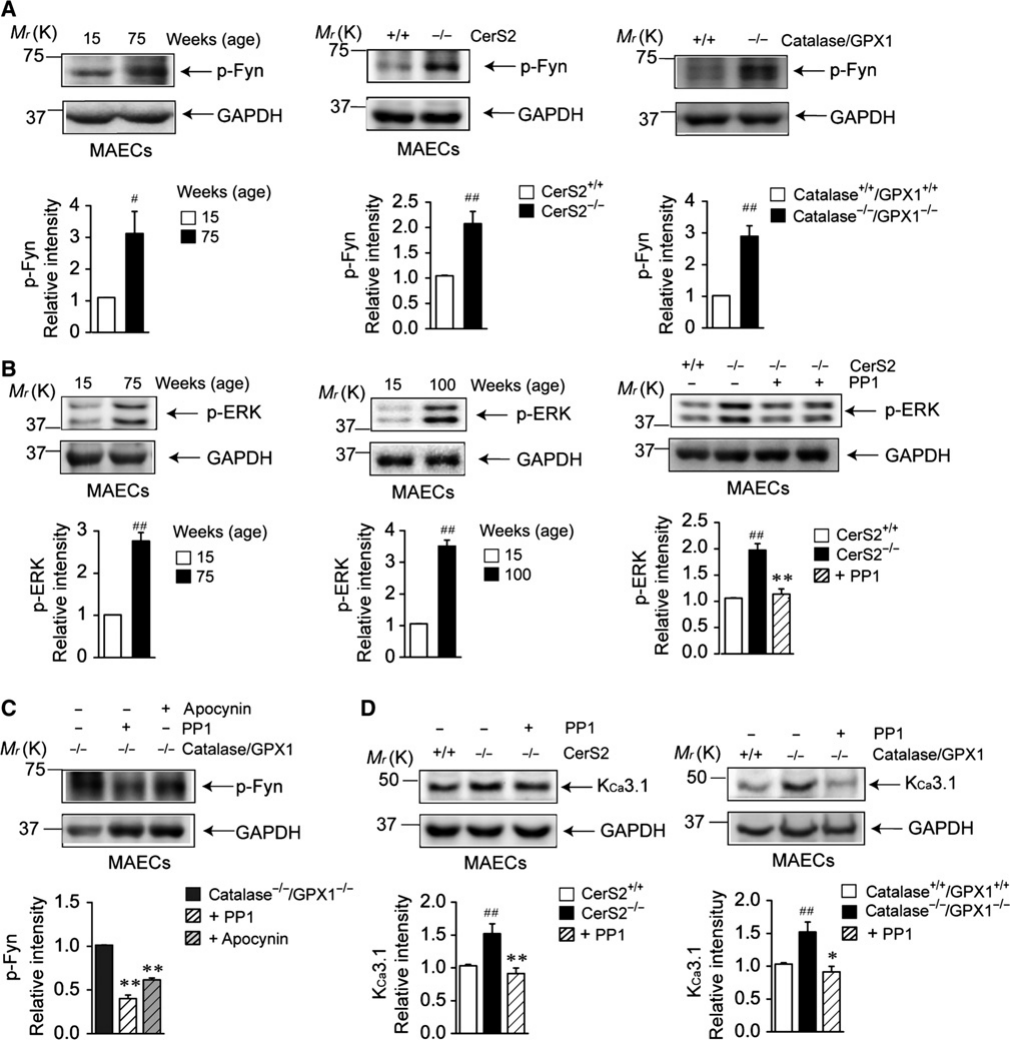
Fyn and ERK is involved in endothelial K_C__a_3.1 upregulation. (A) Protein levels of p‐Fyn in aged vs. young wild‐type, young CerS2 null vs. age‐matched wild‐type, and young catalase^−/−^/GPX1^−/−^ vs. age‐matched wild‐type mouse aortic endothelial cells (MAECs). (B) Protein levels of p‐ERK in aged vs. young wild‐type and CerS2 null vs. age‐matched wild‐type MAECs. Increased p‐ERK levels were reduced by the Fyn inhibitor PP1. (C) Increased p‐Fyn levels were reduced by the NADPH oxidase inhibitor apocynin or by PP1 in catalase^−/−^/GPX1^−/−^ MAECs. (D) K_C__a_3.1 upregulation were reduced by PP1 in CerS2 null and catalase^−/−^/GPX1^−/−^ MAECs. Blots are representatives of four experiments performed with four different cultures. Results were normalized to GAPDH levels. ^*#*^ *P *< 0.05, ^##^ *P *< 0.01 vs. young, age‐matched, or vehicle‐treated wild‐type. *P < 0.05, **P < 0.01 vs. vehicle‐treated CerS2 null or catalase^−/−^/GPX1^−/−^ MAECs.

H_2_O_2_ mediates several intracellular signals as a second messenger and activates the Src family kinase Fyn (Saksena *et al*., 2008). We therefore examined phosphorylated Fyn (p‐Fyn) levels in MAECs. p‐Fyn levels were increased in aged wild‐type, young CerS2 null, and young catalase^−/−^/GPX1^−/−^ MAECs (Fig. 4A). As H_2_O_2_ (Choi *et al*., 2013b) and Fyn (Toni *et al*., 2006) play critical roles in extracellular signal‐regulated kinase (ERK) activation, we examined phosphorylated ERK (p‐ERK) levels in MAECs. In aged wild‐type and young CerS2 null MAECs, p‐ERK levels were increased (Fig. 4B). The increased p‐ERK levels were reduced by 4‐amono‐5‐(4‐methylphenyl)‐7‐(*t*‐butyl)pyrazol[3,4‐*d*]pyrimidine (PP1) in young CerS2 null MAECs (Fig. 4B, right panel). In addition, the increased p‐Fyn levels were reduced by apocynin or by PP1 in catalase^−/−^/GPX1^−/−^ MAECs (Fig. 4C), and the increased K_Ca_3.1 levels were reduced by PP1 in young CerS2 null and catalase^−/−^/GPX1^−/−^ MAECs (Fig. 4D). These results suggest that H_2_O_2_ upregulates K_Ca_3.1 via a Fyn/ERK‐mediated pathway.

### Endothelial K_Ca_3.1 upregulation preserves EDR upon oxidative stress

NADPH oxidase is a major source of superoxide in vascular ECs (Griendling *et al*., 2000). In addition, arginases promote endothelial NO synthase (eNOS) uncoupling through enzymatic competition with the substrate l‐arginine, thereby generating ROS (Yang & Ming, 2013). An increase in ROS generation causes endothelial dysfunction via the functional inactivation of NO. We thus measured levels of NO, arginase 2, and NOX2 in MAECs (Fig. S2). In young CerS2 null MAECs, intracellular NO levels were decreased (Fig. S2A), and levels of arginase 2 and NOX2 were increased (Fig. S2B). In addition, levels of arginase 2 and NOX2 were significantly increased in young catalase^−/−^/GPX1^−/−^ (Fig. S2C) and aged wild‐type (Fig. S2D) MAECs. These results suggest that ROS markedly decrease NO bioavailability in aged wild‐type, young CerS2 null, and young catalase^−/−^/GPX1^−/−^ mice.

**Figure 5:**
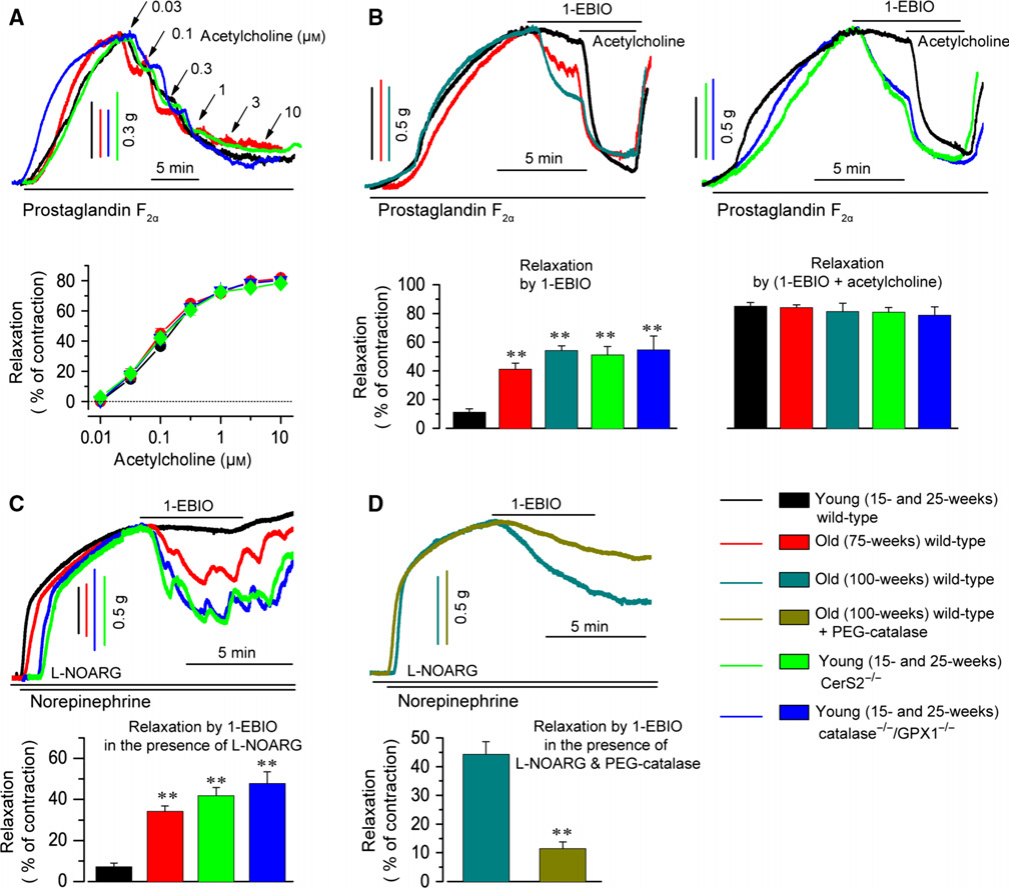
K_C__a_3.1 activation‐induced, *N* ^ω^‐nitro‐l‐arginine (l‐NOARG)‐ and indomethacin‐resistant EDR in aorta. EDR was evoked in aortic rings from young wild‐type, aged wild‐type, young CerS2 null, and young catalase^−/−^/GPX1^−/−^ mice. (A) Acetylcholine induced EDR in a concentration‐dependent manner. (B) The K_C__a_3.1 activator 1‐EBIO‐ and (1‐EBIO + acetylcholine)‐induced EDR was evoked in aortic rings without l‐NOARG pretreatment. (C) Nitric oxide (NO) production was inhibited by l‐NOARG pretreatment, and 1‐EBIO‐induced EDR was evoked. (D) Aortic rings were treated with PEG‐catalase to reduce H_2_O_2_ and K_C__a_3.1 levels, and then 1‐EBIO‐induced EDR was evoked in the presence of l‐NOARG. (A‐D) In each experiment, one aortic ring was obtained from each mouse, and graphs were computed with pooled data from 10 experiments (young wild‐type mice) and four or five experiments (75‐ and 100‐week‐old, young Cers2 null, catalase^−/−^/GPX1^−/−^ mice). The magnitude of maximal relaxation at each treatment was expressed as a percentage of initial prostaglandin F_2α_‐ or norepinephrine‐induced contraction. **P < 0.01 vs. young wild‐type.

We thus examined whether EDR to acetylcholine is reduced in these mice (Fig. 5). As vascular smooth muscle contraction to prostaglandin F_2α_ or norepinephrine was not blunted in these mice, aortic rings from these mice were contracted by prostaglandin F_2α_ or norepinephrine. Precontracted endothelium‐intact aortic rings were relaxed by acetylcholine in a concentration‐dependent manner. Although NO production was significantly reduced in ECs, EDR to acetylcholine was not significantly reduced in aortic rings from aged wild‐type, young CerS2 null, and young catalase^−/−^/GPX1^−/−^ mice (Fig. 5A). In addition, sodium nitroprusside‐induced relaxation of precontracted aortic rings was not changed in these mice (data not shown), suggesting that the reactivity of vascular smooth muscle to NO is not affected. In the presence of indomethacin, EDR to acetylcholine of vascular smooth muscle is evoked by NO released from ECs and by endothelium‐dependent hyperpolarization via endothelial K_Ca_3.1 activation. We therefore compared the magnitude of K_Ca_3.1 activation‐induced EDR in these mice. The K_Ca_3.1 activator 1‐EBIO relaxed precontracted endothelium‐intact aortic rings (Fig. 5B), but did not relax precontracted endothelium‐denuded aortic rings (Fig. S3A), suggesting that K_Ca_3.1 activator‐induced relaxation is endothelium dependent. When the EDR response to the K_Ca_3.1 activator reached a steady state, we added acetylcholine (1 μm) to evoke NO‐induced EDR (Fig. 5B). Compared with young or age‐matched wild‐type mice, 1‐EBIO‐induced EDR was significantly increased, but (1‐EBIO + acetylcholine)‐induced EDR was unchanged, in aged wild‐type, CerS2 null, and catalase^−/−^/GPX1^−/−^ mice. Thus, the ratio of 1‐EBIO‐induced EDR to (1‐EBIO + acetylcholine)‐induced EDR was markedly increased in aged wild‐type, young CerS2 null, and young catalase^−/−^/GPX1^−/−^ mice. As K_Ca_3.1 activation contributes to NO production by increasing intracellular Ca^2+^ levels, we then examined K_Ca_3.1 activator‐induced EDR of endothelium‐intact aortic rings in which NO production was inhibited by pretreatment with *N* ^ω^‐nitro‐l‐arginine (l‐NOARG) (Fig. 5C). Compared with young wild‐type mice, EDR to 1‐EBIO was significantly increased in aged wild‐type, young CerS2 null, and young catalase^−/−^/GPX1^−/−^ mice, indicating that the K_Ca_3.1 contribution to EDR is markedly increased in mice in which endothelial K_Ca_3.1 is upregulated. EDR to NS309 was similar to that to 1‐EBIO (Fig. S3B). As polyethylene glycol‐catalase (PEG‐catalase) reduced K_Ca_3.1 levels in aged MAECs (Fig. S4), we examined whether an increase in K_Ca_3.1 activation‐induced EDR could be reduced by PEG‐catalase in aortas from aged wild‐type mice (Fig. 5D). Treatment with PEG‐catalase markedly reduced 1‐EBIO‐induced EDR in aorta from aged wild‐type mice. These results suggest that reduced EDR to NO can be compensated for by enhancing K_Ca_3.1 activation‐induced EDR in aged wild‐type, young CerS2 null, and young catalase^−/−^/GPX1^−/−^ mice.

### Altering sphingolipid composition changes levels of antioxidant enzymes and upregulates K_Ca_3.1

**Figure 6:**
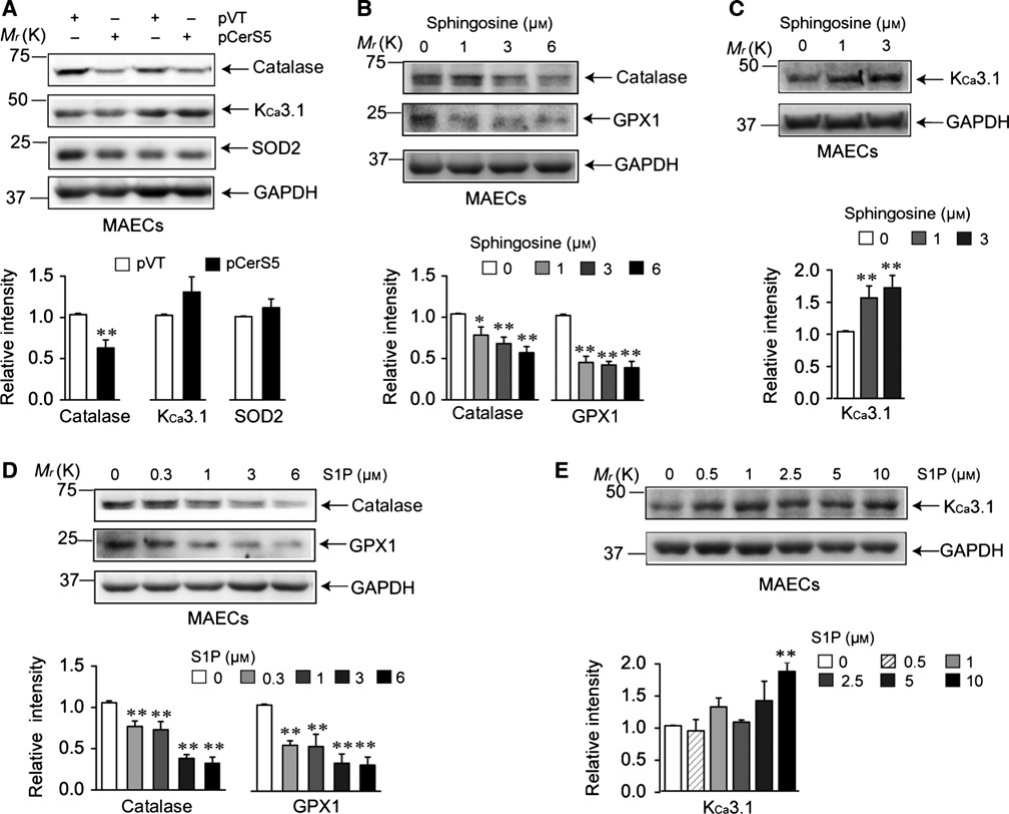
Levels of K_C__a_3.1 and antioxidant enzymes are affected by alteration in sphingolipid composition. After wild‐type mouse aortic endothelial cells (MAECs) were transfected with CerS5 (A) or treated with sphingosine (B,C) or S1P (D,E), protein levels of catalase, GPX1, SOD2, and K_C__a_3.1 were measured. Blots are representatives of three to four experiments performed with three to four different cultures. Results were normalized to GAPDH levels. *P < 0.05, **P < 0.01 vs. wild‐type MAECs transfected with empty vector (A), or treated with vehicle (B–E).

Finally, we examined whether changes in levels of antioxidant enzymes and K_Ca_3.1 are induced by altering sphingolipid composition in young CerS2 null and aged wild‐type MAECs. We determined levels of CerSs and sphingolipids in aortic tissue and MAECs from young CerS2 null mice (Fig. S5). In CerS2 null aortic tissue, the mRNA levels of CerS4‐CerS6 were significantly increased, whereas the levels of CerS1 and CerS3 were not affected (Fig. S5A). The levels of C16‐ and C18‐ceramides were significantly increased, while the levels of C22‐, C24:1‐, and C24‐ceramides were markedly decreased, in CerS2 null aortic tissue (Fig. S5B) and CerS2 null MAECs (Fig. S5C). In addition, the levels of sphingosine, sphingosine 1‐phosphate (S1P), and sphinganine were significantly increased in CerS2 null MAECs (Fig. S5C). We previously reported that the CerS2 ablation‐induced alteration in the levels of CerSs and sphingolipid composition is similar to aging‐related alteration in these measurements in mice (Choi *et al*., 2015b). As CerS5 was upregulated in CerS2 null mice, we examined the effects of CerS5 transfection on levels of antioxidant enzymes and K_Ca_3.1 in wild‐type MAECs (Fig. 6A). CerS5 transfection significantly downregulated catalase without affecting SOD2 levels. K_Ca_3.1 levels were slightly increased, but this increase was not statistically significant. We then examined whether sphingosine or S1P affects K_Ca_3.1 levels because sphingosine or S1P levels were increased in CerS2 null (Fig. S5C) and aged (Choi *et al*., 2015b) mice. Exogenously added sphingosine (Fig. 6B,C) or S1P (Fig. 6D,E) downregulated catalase and GPX1 and upregulated K_Ca_3.1 in a concentration‐dependent manner. Furthermore, exogenously added sphingosine increased H_2_O_2_ generation in wild‐type MAECs (data not shown). These results suggest that altering sphingolipid profile increases K_Ca_3.1 levels by changing levels of antioxidant enzymes.

## Discussion

**Figure 7:**
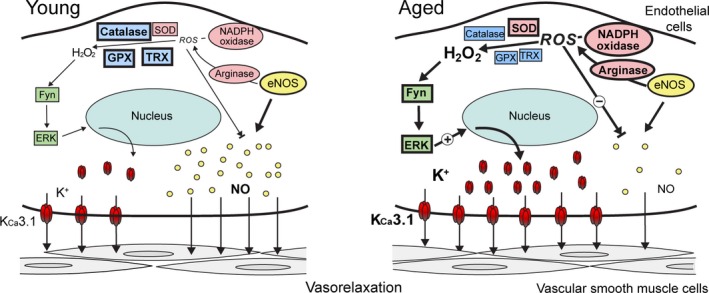
Model for endothelial K_C__a_3.1 upregulation in aging‐related oxidative stress conditions. Sphingolipid composition was altered in aged mice, and altered sphingolipid profile (i.e., increased C16‐sphingolipid, sphingosine, or sphinganine) increased H_2_O_2_ content in mouse aortic endothelial cells by upregulating SOD and downregulating catalase and GPX1. In addition, upregulation of arginase and NADPH oxidase enhanced ROS production. H_2_O_2_ enhanced K_C__a_3.1 expression via a Fyn/ERK‐mediated pathway, and thereby augmented K_C__a_3.1 activation‐induced EDR. In contrast, enhanced ROS production reduced NO bioavailability, and thereby might impair NO‐induced EDR. Thus, enhanced expression of K_C__a_3.1 compensates for diminished EDR to NO during aging‐related oxidative stress. SOD, superoxide dismutase; GPX1, glutathione peroxidase 1; ROS, reactive oxygen species; EDR, endothelium‐dependent relaxation; NO, nitric oxide.

The results of our study show that alteration in sphingolipid acyl chain length and composition upregulates SOD and downregulates catalase and GPX1 in aged wild‐type and young CerS2 null mice, thereby increasing H_2_O_2_ content (Fig. 7). The increased ROS production impairs endothelial NO production and NO‐induced EDR of vascular smooth muscle. On the other hand, H_2_O_2_ induces endothelial K_Ca_3.1 upregulation, which enhances K_Ca_3.1 activation‐induced EDR, thereby maintaining EDR to acetylcholine. These findings represent the first evidence of a compensatory role for endothelial K_Ca_3.1 in mediating aortic vasorelaxation during old age and under conditions of oxidative stress, which may be implicated in age‐associated cardiovascular disorders.

Among ROS, H_2_O_2_ levels were increased in ECs of aged wild‐type and young CerS2 null mice. This notion is supported by three observations; firstly, fluorescence from the H_2_O_2_‐sensitive dyes, CM‐DCFH‐DA and peroxy‐orange 1, was markedly increased in MAECs from aged wild‐type and young CerS2 null mice. Secondly, catalase and GPX1, which degrade H_2_O_2_, were downregulated, and SOD, which generates H_2_O_2_ from superoxide, was upregulated. Thirdly, p‐Fyn and K_Ca_3.1, which are positively regulated by H_2_O_2_, were upregulated in ECs of aged wild‐type and young CerS2 null mice. ROS‐generating enzymes, such as NADPH oxidases, xanthine oxidase, and NO synthases, are activated by ceramide (Lecour *et al*., 2006). In addition, arginase upregulation, found in young CerS2 null and aged wild‐type mice, might contribute to increased oxidative stress, as eNOS produces superoxide via eNOS uncoupling caused by arginases. Thus, downregulation of catalase and GPX1 and upregulation of SOD contribute to increased H_2_O_2_ levels, which is consistent with the enhanced H_2_O_2_ availability and the decreased catalase activity in the microvascular endothelium of aged mice (Socha *et al*., 2015).

K_Ca_3.1 upregulation in young catalase^−/−^/GPX1^−/−^ mice suggests that changes in the levels of antioxidant enzymes, including downregulation of catalase and GPX1, play a critical role in K_Ca_3.1 upregulation in aged wild‐type and young CerS2 null mice. The presence of NADPH oxidase subunits or cofactors and antioxidant enzymes in lipid rafts (Li & Zhang, 2013) suggests that lipid rafts play an important role in the regulation of these enzymes. In addition, alteration of lipid rafts was suggested to occur in CerS2 null mice (Park *et al*., 2013), and the function of lipid rafts is altered with aging (Fulop *et al*., 2012). Thus, changes in lipid rafts might cause such alterations in the levels of antioxidant enzymes in aged wild‐type and CerS2 null mice.

As sphingolipids are important structural component of membranes, altering the sphingolipid composition might cause changes in lipid rafts. Changes in the levels of antioxidant enzymes were induced in CerS2‐null mice in which sphingolipid composition was altered. In addition, CerS5 transfection or exogenously added sphingosine or S1P changed the levels of antioxidant enzymes in MAECs. These results suggest that alterations in ceramide composition or the content of sphingosine or S1P play an important role in changing these levels. We therefore suggest that changes in the levels of antioxidant enzymes and resultant K_Ca_3.1 upregulation in MAECs from aged wild‐type and young CerS2 null mice are caused by an altered sphingolipid profile (i.e., increased C16‐sphingolipid, decreased C22‐C24‐sphingolipid, or increased long chain bases such as sphingosine or sphinganine).

Studies have shown that H_2_O_2_ enhances the tyrosine phosphorylation of Src kinases and mitogen‐activated protein kinases, leading to activation of gene expression, including activator protein‐1 (Hardwick & Sefton, 1997; Jaramillo & Olivier, 2002; Gaitanaki *et al*., 2003). Consistent with these results, H_2_O_2_ increased the phosphorylation of the Src family kinase Fyn. H_2_O_2_ and Fyn play critical roles in ERK activation (Toni *et al*., 2006; Choi *et al*., 2013b). As K_Ca_3.1 synthesis occurs via an ERK/activator protein‐1‐mediated pathway, K_Ca_3.1 synthesis might be induced via a H_2_O_2_/Fyn/ERK‐mediated pathway. However, as the expression of membrane proteins in the plasma membrane is determined by a balance between synthesis/forward trafficking from the endoplasmic reticulum, endocytosis, and recycling/degradation, further studies are required to clarify the effects of altering the sphingolipid composition on these processes.

There is now a wealth of evidence suggesting that oxidative stress is a major cause of endothelial dysfunction (Gori & Munzel, 2011). However, signaling molecules of the ROS pathway, such as H_2_O_2_, participate in the modulation of various cellular activities including cell proliferation, differentiation, and apoptosis (Sies, 2014). Our results clearly showed that H_2_O_2_ affected endothelial function by increasing K_Ca_3.1 expression. As consistent with previous evidence in intact microvascular endothelial tubes (Behringer *et al*., 2013), H_2_O_2_ activated K_Ca_3.1 currents in aortic ECs (Choi *et al*., 2013b). Endothelial dysfunction, which was manifested in the form of diminished NO bioavailability, was well compensated for by an increased expression of K_Ca_3.1 protein, as shown in aged wild‐type, CerS2 null, and catalase^−/−^/GPX1^−/−^ mice, and an upregulation of K_Ca_3.1 channel activity.

The K_Ca_3.1 activator‐induced relaxation resistant to l‐NOARG and indomethacin might be mediated by K_Ca_3.1 activation‐induced endothelial hyperpolarization. This suggestion is supported by the following observations: K_Ca_3.1 activator‐induced relaxation response was completely absent after denudation of the endothelium, demonstrating its dependency on the presence of intact ECs. The absence of the relaxation effect of the K_Ca_3.1 activators on vascular smooth muscle can be explained by the absence of K_Ca_3.1 in vascular smooth muscle cells. In addition, the portion of the relaxation resistant to l‐NOARG and indomethacin was induced by the K_Ca_3.1 activators 1‐EBIO or NS309, suggesting that this portion of EDR is mediated through K_Ca_3.1 activation. Furthermore, the portion of the relaxation resistant to l‐NOARG and indomethacin was enhanced in the aortic rings in which endothelial K_Ca_3.1 was upregulated, suggesting that the magnitude of the resistant part of EDR is closely related with K_Ca_3.1 levels.

The K_Ca_3.1 activator 1‐EBIO or NS309 also activates K_Ca_2.3 with similar potency (Coleman *et al*., 2014). K_Ca_2.3 shares many properties with K_Ca_3.1 (Jensen *et al*., 2001) and it has been implicated in endothelium‐dependent dilation (Grgic *et al*., 2009). In addition, it was found that mRNA and protein levels of K_Ca_2.3 were significantly increased in MAECs or aortic tissues from aged wild‐type and CerS2 null mice (Fig. S6). These results suggest that K_Ca_2.3 activation‐induced endothelial hyperpolarization contributes to 1‐EBIO‐ or NS309‐induced relaxation resistant to l‐NOARG and indomethacin. Further studies are required to investigate the mechanisms underlying K_Ca_2.3 upregulation in MAECs from aged wild‐type and CerS2 null mice.

Endothelium‐dependent relaxation induced by endothelial hyperpolarization or endothelium‐derived hyperpolarizing factor is more important in the smaller arteries than in the large conductance arteries (Sandow & Hill, 2000). In agreement, K_Ca_3.1 activator‐induced EDR resistant to l‐NOARG was negligible in aortas from young wild‐type mice. When increased oxidative stress impaired NO bioavailability and l‐NOARG‐sensitive EDR in aged wild‐type and CerS2 null mice, K_Ca_3.1 contributed to the maintenance of endothelial vasodilator function by enhancing endothelium‐dependent hyperpolarization‐mediated vasodilation. A similar enhancement of EDR, which is resistant to l‐NOARG and indomethacin, and a similar contribution of K_Ca_3.1 and/or K_Ca_2.3 to EDR were reported to occur in arteries from rats with obesity (Chadha *et al*., 2010; Climent *et al*., 2014), type 2 diabetes mellitus (Schach *et al*., 2014), and spontaneous hypertension (Giachini *et al*., 2009; Simonet *et al*., 2012).

The present study has some limitations that need to be acknowledged. First, EC phenotypes might be changed after isolation and culture, because K_Ca_1.1 upregulation (Sandow & Grayson, 2009) and K_Ca_3.1 downregulation (data not shown) often occur in cultured ECs. Nevertheless, EC isolation and culture is necessary to examine the levels of proteins such as antioxidants in ECs, because these proteins are expressed not only in ECs but also in vascular smooth muscle cells. In addition, cultured ECs are necessary to examine the mechanism by which expression levels of K_Ca_3.1 or K_Ca_2.3 are modulated. As expression levels of K_Ca_3.1 and K_Ca_2.3 in MAECs are well maintained within 2 passages, we used MAECs within 2 passages in the present study. Aging‐related increases in K_Ca_2.3 expression levels were similar in aortic tissues and cultured MAECs within two passages (Fig. S6C,D). Second, compared with conduit arteries such as aorta, resistant arteries and arterioles play more important roles in maintaining vascular homeostasis by regulating total peripheral resistance and blood flow. Thus, small arteries such as branches of superior mesenteric arteries might be more suitable than aorta for the present study. However, it is not practical (using current techniques) to obtain enough ECs within two passages for molecular determinations using small arteries from mice. In contrast, we could obtain enough ECs within two passages for one molecular experiment using aortas.

In conclusion, our data suggest that alteration in sphingolipid acyl chain length and composition induces ROS, especially H_2_O_2_, generation by changing the levels of antioxidant enzymes and thereby causes endothelial dysfunction as manifested by reduced NO bioavailability (Fig. 7). On the other hand, H_2_O_2_ induces K_Ca_3.1 upregulation via the H_2_O_2_/Fyn/ERK‐signaling pathway, and thereby compensates for a diminished NO‐induced EDR response by enhancing K_Ca_3.1 activation‐induced EDR. To the best of our knowledge, this is the first study to demonstrate the signaling mechanisms underlying a compensatory role for endothelial K_Ca_3.1 in mediating vasorelaxation during old age and under conditions of oxidative stress. Such plasticity within the EDR mechanisms presents a significant potential target for therapeutic intervention.

## Experimental procedures

For description of measurement of intracellular ROS, measurement of intracellular NO, Western blotting, PCR, transfection, LC‐MS/MS analysis of SLs, and chemicals, please refer the Appendix S1.

### Animals

CerS2 null mice were generated as described (Pewzner‐Jung *et al*., 2010), and catalase^−/−^/GPX1^−/−^ mice were generously donated by Dr. Ye‐Shih Ho (Wayne State Medical School, Detroit, MI) (Johnson *et al*., 2010). We studied CerS2 null mice (15‐ or 25‐week‐old; *n *= 120) and age‐matched wild‐type (F1 of 129S4/SvJae × C57BL/6) mice (*n *= 85), GPX1^−/−^/catalase^−/−^ mice (15‐ or 25‐week‐old; *n* = 22) and age‐matched C57BL/6 wild‐type mice (*n* = 18), and aged (75‐ and 100‐week‐old; *n* = 25 and 18, respectively) and young (15‐week‐old; *n* = 30) C57BL/6 wild‐type mice. In all mice types, 15‐ or 25‐week‐old mice were classified as young, and 75‐ or 100‐week‐old mice were as aged. Mice were anesthetized by an intraperitoneal injection of pentobarbital sodium (50 mg kg^−1^ body weight). The investigation was approved by the local ethics committee, the Institutional Review Board of the Ewha Womans University, and was in accordance with the Declaration of Helsinki, the Animal Care Guidelines of the Ewha Womans University, Medical School, and the National Institutes of Health Guide for the Care and Use of Laboratory Animals.

### Cell isolation and culture

All mice were genotyped using polymerase chain reaction. The mice were fed with an autoclaved diet and water *ad libitum*. MAECs were isolated from the mouse aortas as described (Choi *et al*., 2015a). Briefly, periadventitial fats and connective tissues around the aorta were carefully cleaned in Ca^2+^‐free phosphate‐buffered saline under a dissecting microscope. Matrigel (BD Biosciences, San Jose, CA, USA) was plated and polymerized at 37 °C for 30 min. After that, aorta pieces were placed with the intima side down on the Matrigel. To demonstrate the endothelial nature of the cell, 1,1′‐dioctadecyl‐3,3,3′,3′‐tetramethyl‐indocarbocyanine perchlorate‐labeled acetylated low‐density lipoprotein (Biomedical Technologies Inc., Stoughton, MA, USA) uptake assay was employed. MAECs were used within two passages and not above three passages. Each time we isolated and cultured MAECs, the thoracic aortas were dissected out from two or three mice. MAECs cultured from each aorta were pooled together and used in each experiment.

### Contraction measurement on isolated aortic rings

Mice were anesthetized by an intraperitoneal injection of pentobarbital sodium (50 mg kg^−1^ body weight). The thoracic aorta was dissected out and cut into rings of about 1 mm. Mechanical responses were recorded from the aortic ring segments using a custom myograph. Each aortic ring was threaded with two strands of tungsten wire (120 μm in diameter). One wire was anchored in the organ bath chamber (1 mL) and the other was connected to a mechanotransducer (Grass, FT‐03) mounted on a three dimensional manipulator. Optimal resting tension (0.6–0.8 g) was applied. The muscle chamber was perfused at a flow rate of 2.5 ml min^−1^ with oxygenated (95% O_2_/5% CO_2_) Krebs/Ringer bicarbonate solution with a peristaltic pump. The composition (in mm) of the Krebs solution was NaCl 118.3, KCl 4.7, MgCl_2_ 1.2, KH_2_PO_4_ 1.22, CaCl_2_ 2.5, NaHCO_3_ 25.0, glucose 11.1, pH 7.4.

### Statistical analysis

Data represent the mean ± SEM of the experiments performed with aortas or MAECs. MAECs were isolated and cultured from aortas of two (young) or three (aged) mice at a time. MAECs cultured from each aorta were pooled together and used in each experiment (*n* = 1). To measure the strength of a contraction, one aortic ring was obtained from each mouse (*n* = 1), and graphs were computed with pooled data from 4 to 10 experiments. To examine the statistical significance between groups, one‐way analysis of variance (ANOVA) with Bonferroni's *post hoc* or two‐tailed Student's *t*‐test was used. A *P* value of 0.05 or lower was considered statistically significant. Calculations were performed with spss 14.0 for Windows (SPSS, Chicago, IL, USA).

## Funding

This research was supported by Basic Science Research Program through the Nation Research Foundation of Korea funded by the Ministry of Education, Science and Technology (NRF‐2013R1A1A2010851 & NRF‐2013R1A1A2064543).

## Author contributions

S Choi and SH Suh performed study concept and design, obtained funding. S Choi, JA Kim, H Li, and KO Shin performed experiments; S Choi, GT Oh, YM Lee, S Oh, and SH Suh performed analysis and interpretation of data; Y Pewzner‐Jung generated CerS2 null mice; S Choi, Y Pewzner‐Jung, AH Futerman, and SH Suh contributed to writing the manuscript.

## Conflict of interests

None declared.

## Supporting information


**Appendix S1** Supplementary materials and methods.
**Fig. S1** Changes in levels of TRXs in MAECs from CerS2 null mice.
**Fig. S2** Changes in levels of NO, ARG2 and NOX2 in MAECs from CerS2 null, catalase^−/−^/GPX^−/−^, and aged wild‐type mice.
**Fig. S3** l‐NOARG‐ and indomethacin‐resistant EDR by NS309.
**Fig. S4** PEG‐catalase reduced KCa3.1 levels in aged MAECs.
**Fig. S5** Changes in the levels of CerS and SLs in aorta or MAECs from CerS2 null mice.
**Fig. S6** Changes in expression levels of KCa2.3 in MAECs or aorta from CerS2 null, and aged wild‐type mice.
