# Supplementary material for: KCa3.1 upregulation preserves endothelium‐dependent vasorelaxation during aging and oxidative stress
Source: Aging Cell. 2016 Jun 30;15(5):801–10. doi: 10.1111/acel.12502 (PMC5013018; doi:10.1111/acel.12502)

SUPPORTING INFORMATION

SUPPLEMENTARY MATERIALS AND METHODS

Measurement of intracellular ROS

Primary cultured MAECs plated on 96-well microplates (for direct quantification) or on microscopic coverslips (for fluorescent images) were incubated in the superoxide-sensitive dye DHE (10 μM) or the H_2O_2-sensitive dye CM-DCFH-DA (10 μM) or peroxy-orange1 (5 μM) for 20 minutes, as described (Engelmann *et al.* 2005; Rhee *et al.* 2010; Dickinson & Chang 2011). Samples were read directly using a microplate fluorescence reader (model SpectraMax, Molecular Devices, Sunnyvale, CA) or detected by confocal laser microscopy (model LSM 510, Carl Zeiss, Germany). Excitation/emission wavelengths employed for detection of respective fluorescent dyes in 96-well microplate reader are as follows: CM-DCFH-DA (ex 488 nm/em 520 nm), DHE (ex 518 nm/em 605 nm), peroxy-orange1 (ex 540 nm/em 585 nm). Confocal laser-scanning microscopic images for CM-DCFH-DA were acquired in an excitation wavelength of 488 nm, a long path emission filter (505 to 540 nm), and a Zeiss C-Apochromat 40× objective with a 10× eyepiece. Confocal laser-scanning microscopic images of DHE were acquired in an excitation wavelength of 543 nm, a rhodamine emission filter, and a Zeiss Plan-Apochromat 63× oil immersion objective with a 10× eyepiece. Fluorescent intensities acquired from confocal laser-scanning microscopy were quantified by image processing software Image J (http://imagej.nih.gov/ij).

Measurement of intracellular nitric oxide (NO)

To monitor quantitative changes in NO production from primary MAECs, the NO fluorescence

detection probe kit (Enzo Life Sciences, Farmingdale, NY) was used according to the manufacturer's instruction (Wardman 2007). Briefly, MAECs plated on 96-well microplates were incubated under normal cell-culture conditions with non-fluorescent cell-permeable NO detection dye (10 μM) for 20 min that react with NO in the presence of oxygen with high specificity and accuracy, yielding a red fluorescent product. Levels of fluorescence were measured by using a filter set of 643 nm (ex)/670 nm (em) in fluorescence reader (model SpectraMax, Molecular Devices, Sunnyvale, CA).

Western Blotting

Cell lysate samples were used. Total protein was measured using the bicinchoninic acid assay (Pierce Biotechnology, Rockford, IL). Protein was separated by SDS-PAGE on 7.5–12% gels and transferred to nitrocellulose membranes. The membrane was blocked using 5% BSA in Tris-buffered saline containing 0.1% Tween-20 (TBST) at room temperature for 1 hour. The primary antibody was diluted in TBST containing 5% BSA and incubated with the membrane overnight at 4°C. The primary antibodies used in this study were anti-arginase 2 (sc-20151, Santa Cruz Biotechnology, Santa Cruz, CA), anti-catalase (ab16731, Abcam, Cambridge, MA), anti-GPX1 (ab22604, Abcam), anti-K_{Ca}3.1 (sc-32949, Santa Cruz Biotechnology), anti-NOX2 (ab31092, Abcam). p-ERK (#9101, Cell Signaling Technology, Beverly, MA), anti-p-Fyn (07-909, EMD Millipore, Darmstadt, Germany), anti-SOD1 (sc-11407Santa Cruz Biotechnology), anti-SOD2 (sc-30080, Santa Cruz Biotechnology), anti-K_{Ca}2.3 (sc-28621, Santa Cruz Biotechnology), anti-GAPDH (AB2302, EMD Millipore), anti-β-actin (sc-130656, Santa Cruz Biotechnology). After washing 4 times for 5 minutes, membranes were incubated with the secondary antibody in TBST containing 5% BSA for 1 hour at room temperature. The bands were visualized by

chemiluminescence analysis (GE Healthcare, Piscataway, NJ). Data processing was performed using a luminescent image analyzer LAS-3000 (Fujifilm, Tokyo, Japan) and IMAGE GAUGE software.

Polymerase chain reaction (PCR)

RNA was isolated from the cells using the RNeasy Mini Kit (Qiagen Inc, Valencia, CA), and then reverse transcribed using a High Capacity cDNA Archive Kit (Applied Biosystems, Foster City, CA). PCR was performed on PCR Thermal Cycler (BioRad, Hercules, CA) or ABI 7000 sequence detection system (Applied Biosystems) using a SYBR Green PCR Master Mix (Applied Biosystems). The primers used were listed in **Table 1**.

Table 1 Primers used in current study.

**Gene**	**Sequence**
CerS1	Sense 5′-AGT CTG TGC CTG ACA TTC CG-3′
	Anti-sense 5′-GCC ATT CCT CAG TGG CTT CT-3′
CerS2	Sense 5′- GCT GGA GAT TCA CGT ATT AC-3′
	Anti-sense 5′- GAA CAC AAT GAA GAG GTT GT-3′
CerS3	Sense 5′- GAG CGC CAG GTT GAA AGA TG-3′
	Anti-sense 5′- TAC TGG GAC GGC AGC AAA G-3′
CerS4	Sense 5′- CCG TGG GAC TGA TAG GCT TC-3′
	Anti-sense 5′- CGT GTA GAT GAC CTG GGT GG-3′
CerS5	Sense 5′- CAA CTG GAC TGG AGT GTT CG-3′
	Anti-sense 5′- TCT CGA GAG TGG CTG ATA CG-3′
CerS6	Sense 5′- CTG GAC TGG GAT GTT CGG AG-3′
	Anti-sense 5′- CAG CTG TGA GTG GCT GGT AA-3′
Thioredoxin1	Sense 5′- GCG CTC CGC CCT ATT TCT AT-3′
	Anti-sense 5′- TCA CCA TTT TGG CTG TTG CG-3′
Thioredoxin2	Sense 5′- TGG GCT TCC CTC ACC TCT AA-3′
	Anti-sense 5′- GGC GAC CAT CTT CTC TAG CC-3′
$K_{Ca}2.3$	Sense 5′- GGA GGG CCC TGT TTG AAA AG-3′
	Anti-sense 5′- TCA GGG CCA ACG AAA ACA TG-3′
GAPDH	Sense 5′- CTC CCA CTC TTC CAC CTT CG-3′
	Anti-sense 5′- TAG GGC CTC TCT TGC TCA GT-3′

Transfection

MAECs were transiently transfected for 24 hours with CerS5 plasmid using JET-PEI (Polyplus-Transfection Inc, New York, NY) or Effectene (Qiagen Inc, Valencia, CA), according to the manufacturer's suggested protocol.

LC-MS/MS analysis of sphingolipids

Ceramides were extracted with the mixture of 50 µL cell lysate (50 µg) or 10 mg tissue, 100 pM internal standard C_{17}-ceramide (d17:1/C18:0), and 2 mL chloroform/methanol (2:1, v/v). Sphingosine, S1P and sphinganine were extracted with the mixture of 50 µL cell lysate (50 µg) or 10 mg tissue, 100 pM internal standard C_{17}-sphingosine and C_{17}-S1P, 1.2 mL chloroform/methanol (2:1, v/v) and 0.3 mL 0.1N HCl. The lower phase was collected after repeated extraction and dried under a vacuum. The resulting residue was re-dissolved in 100 µL methanol, and 10 µL was then injected into the LC-ESI-MS/MS machine. For optimization, a mixture of ceramide standards or S1P and sphingosine was infused directly into the mass spectrometer and all source parameters and ionization conditions were adjusted to improve the sensitivity of the assay. Extracted samples (10 µL) were injected into an HPLC (Agilent 1200 series, Agilent, CA) and separated on a reverse phase KINETEX C18 column (2.1 × 50 mm, ID: 2.6 µm) (Phenomenex, St. Louis, MO). Ceramides were resolved using a linear gradient starting from 8% mobile phase A (water containing 0.2% formic acid) at a flow rate of 0.3 mL/min for 1 minute, to 100% mobile phase B (methanol containing 0.2% formic acid) over 3 minutes, followed by 100% mobile phase B for 12 minutes. The column was then equilibrated for 8 minutes with 92% mobile phase B. Sphingosine, S1P and sphinganine were resolved using a linear gradient from 50% mobile phase A (water containing 0.2% formic acid with 1 mM

ammonium formate) at a flow rate of 0.3 mL/min for 1 minute, to 100% mobile phase B (methanol containing 0.2% formic acid with 1 mM ammonium formate) over 3 minutes, followed by 100% mobile phase B for 8 minutes. The column was then equilibrated for 7 minutes with 50% mobile phase B. The HPLC column effluent was introduced into an API 3200 Triple quadrupole mass spectrometer (ABCIEX, Toronto, Canada) and analyzed using electrospray ionization in the positive mode. Analyses were performed using electrospray ionization in the positive-ion mode with multiple reaction monitoring to simultaneously select both the parent and the characteristic daughter ions specific to each analyte from a single injection. The MS/MS transitions (m/z) were 510→264 for C14, 538→264 for C16, 552→250 for C17, 566→264 for C18, 594→264 for C20, 648→264 for C24:1, 650→264 for C24, 366→250 for C_{17}-S1P, 380→264 for C_{18}-S1P, 286→238 for C_{17}-sphingosine, 300→252 for C_{18}-sphingosine, 302→60 for C_{18}-sphinganine. Data were acquired using Analyst 1.4.2 software. Levels of sphingolipids were measure by LC-ESI-MS/MS (API 3200 Triple quadruple mass, ABCIEX, Framingham, MA, USA). Data were acquired using Analyst 1.4.2 software (Life Technologies/Applied Biosystems, Foster City, CA).

Chemicals

All agents were obtained from Sigma-Aldrich (St Louis, MO) unless indicated otherwise. Cell-permeable fluorescence dye CM-DCFH-DA, or peroxy-orange1 (Tocris Bioscience, London, United Kingdom) was used for the detection of H_2O_2 levels, and DHE (Invitrogen™, Eugene, OR) was used for the detection of superoxide. To neutralize H_2O_2, cells were treated with catalase (1000 or 2000 units) for 24 hours or PEG-catalase (30 units) for 12 hours. ROS levels were decreased by applying the antioxidants (tiron (50 μM), tempol (5 μM) or NAC (10 mM)) or

the NADPH oxidase inhibitor apocynin (100 μM) for 24 hours. Superoxide levels were increased in cells by treatment with the SOD inhibitor 2-ME (15 or 50 μM) for 24 hours. The Src family kinase Fyn, which is activated by H_2O_2, was inhibited by the treatment with the Fyn inhibitor PP1 (100 nM) or catalase for 24 hours. Cells were treated with sphingosine or S1P for 24 hours. Aortic rings were precontracted with prostaglandin $F_{2\alpha}$ (1 μM) or norepinephrine (1 μM) and EDR was induced by acetylcholine. L-NOARG (30 μM for 1 hour) was used to inhibit NO production. $K_{Ca}3.1$ activation-induced EDR was induced by the $K_{Ca}3.1$ activator 1-EBIO (100 μM) or NS309 (200 nM). In all experiments to measure EDR, indomethacin (10 μM) was present in the Krebs buffer to inhibit prostacyclin production. 1-EBIO, 2-ME, apocynin, CM-DCFH-DA, DHE, peroxy-orange1, NS309, prostaglandin $F_{2\alpha}$, PEG-catalase, and PP1 were dissolved in dimethyl sulfoxide (DMSO). Catalase, indomethacin, L-NOARG, NAC, norepinephrine, tiron, and tempol were dissolved in distilled water. Sphingosine or S1P was dissolved in chloroform or methanol. The final concentration of DMSO, chloroform or methanol in media was less than 0.1% and it did not have any effect on the activities tested in this study (data of DMSO chloroform or methanol effect are not shown in this study).

Ceramides (acyl chain lengths of C14, C16, C18, C22, C24, and C24:1), C17-ceramide (d17:1/C18:0), sphingosine, S1P, sphinganine and C_{17}-sphingosine, C_{17}-S1P as an internal standard were obtained from Avanti Polar Lipids (Alabaster, AL). All other solvents and chemicals for sphingolipid extraction and LC-MS/MS analysis either high performance liquid chromatography grade from Merck (Darmstadt, Germany) or analytical purity were from sigma-Aldrich (St. Luis,MO, USA).

References

Dickinson BC, Chang CJ (2011) Chemistry and biology of reactive oxygen species in signaling or stress responses. *Nat Chem Biol*. **7**, 504-511.

Engelmann J, Volk J, Leyhausen G, Geurtsen W (2005) ROS formation and glutathione levels in human oral fibroblasts exposed to TEGDMA and camphorquinone. *J Biomed Mat Res B: Appl Biomater*. **75B**, 272-276.

Rhee SG, Chang TS, Jeong W, Kang D (2010) Methods for detection and measurement of hydrogen peroxide inside and outside of cells. *Mol Cells*. **29**, 539-549.

Wardman P (2007) Fluorescent and luminescent probes for measurement of oxidative and nitrosative species in cells and tissues: progress, pitfalls, and prospects. *Free Radic Biol Med*. **43**, 995-1022.

Supplemental Figure Legends & Figures

Fig. S1. Changes in levels of TRXs in MAECs from CerS2 null mice.

mRNA levels of TRX1 and TRX2 were measured in MAECs from 25-week-old CerS2 null and age-matched wild-type mice. Results were normalized to GAPDH levels. Data represent the mean ± SEM of three experiments performed with three different cultures. ***P* < 0.01 versus age-matched wild-type MAECs.

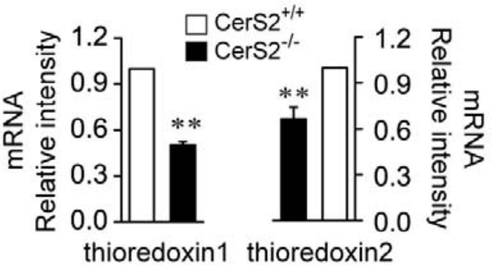

Fig. S2. Changes in levels of NO, ARG2 and NOX2 in MAECs from CerS2 null, catalase$^{-/-}$/GPX$^{-/-}$, and aged wild-type mice.

(A) NO content in MAECs from 25-week-old CerS2 null and age-matched wild-type mice. **(B-D)** Protein levels of ARG2 and NOX2 were determined in MAECs from CerS2 null and age-matched wild-type mice **(B)**, in MAECs from 25-week-old catalase$^{-/-}$/GPX1$^{-/-}$ and age-matched wild-type mice **(C)**, or aged (75-week-old) and young (15-week-old) wild-type mice **(D)**. Data represent the mean ± SEM of three experiments performed with three different cultures. Results were normalized to GAPDH or β–actin levels. $^{\#}P < 0.05$, $^{**}P < 0.01$ versus age-matched wild-type MAECs.

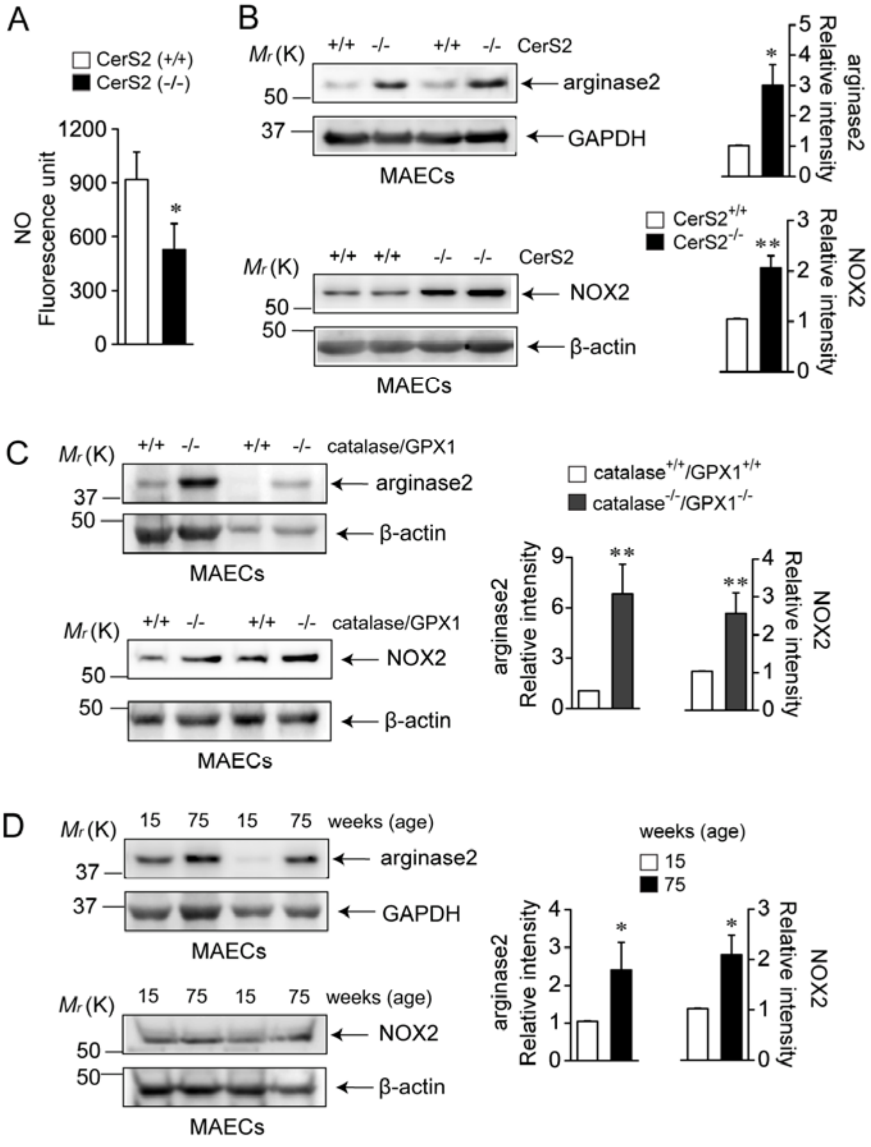
A
CerS2 (+/+)
CerS2 (-/-)
NO
Fluorescence unit
B
Mr (K)
+/+ -/- +/+ -/- CerS2
arginase2
GAPDH
MAECs
arginase2
Relative intensity
CerS2+/+
CerS2-/-
+/+ +/+ -/- -/- CerS2
NOX2
β-actin
NOX2
C
+/+ -/- +/+ -/- catalase/GPX1
catalase+/+/GPX1+/+
catalase-/-/GPX1-/-
D
15 75 15 75 weeks (age)
weeks (age)
15
75

Fig. S3. L-NOARG- and indomethacin-resistant EDR by NS309.

EDR was evoked in aortic rings from young wild-type, aged wild-type, CerS2$^{-/-}$, and catalase$^{-/-}$/GPX1$^{-/-}$ mice. Indomethacin was used to inhibit prostacyclin production. **(A)** Effects of 1-EBIO and NS309 on aortic strips with denuded endothelium. Endothelium of aortic strips was removed by gentle rubbing with a cotton ball. **(B)** The $K_{Ca}3.1$ activator NS309- and (NS309+ACh (1 μM))-induced EDR was evoked in aortic rings without L-NOARG pretreatment. **(C)** NO production was then inhibited by L-NOARG pretreatment and NS309-induced EDR was evoked. **(B,C)** Bar graphs were computed with pooled data from 10 experiments (young wild-type mice) and 4 or 5 experiments (75- and 100-week-old, young Cers2 null, catalase$^{-/-}$/GPX1$^{-/-}$ mice). The magnitude of maximal relaxation at each treatment was expressed as a percentage of initial prostaglandin $F_{2\alpha}$- or norepinephrine-induced contraction. **$P < 0.01$ versus young wild-type.

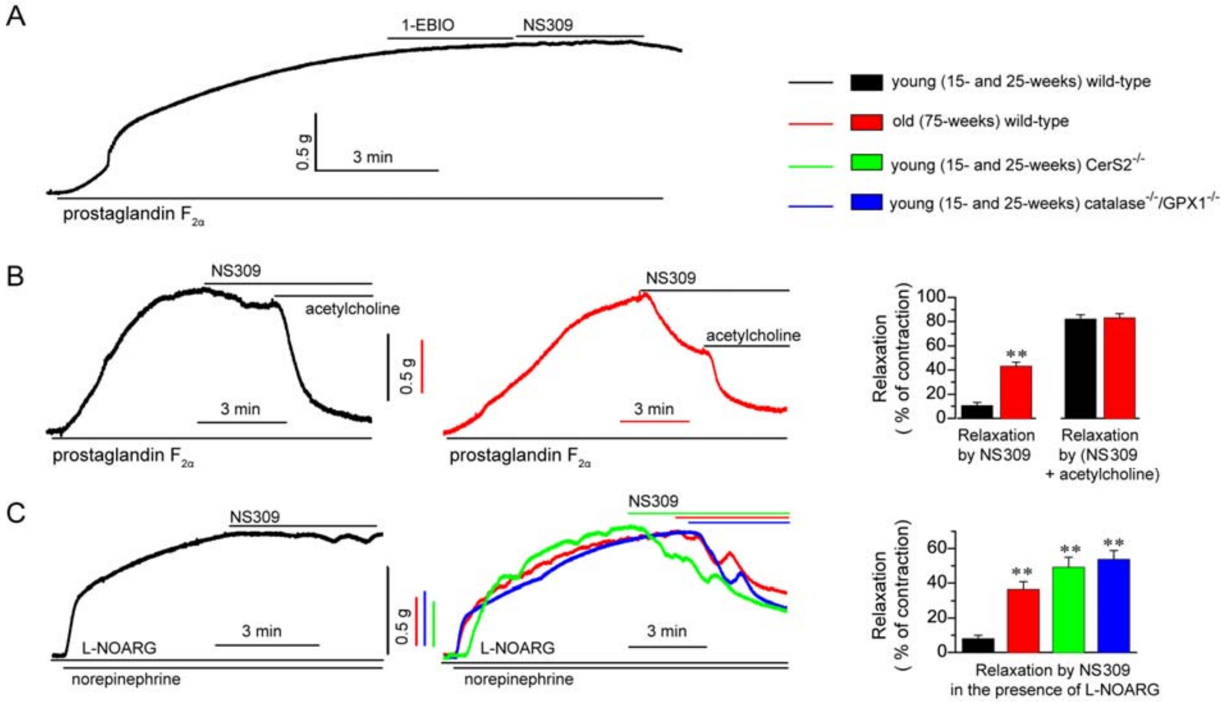

Fig. S4. PEG-catalase reduced $K_{Ca}3.1$ levels in aged MAECs.

MAECs from aged wild-type mice were treated with PEG-catalase and protein levels of $K_{Ca}3.1$ were examined. Bar graph was made with pooled data from four experiments performed with four different cultures. Results were normalized to GAPDH levels. **P < 0.01 versus vehicle treated aged wild-type MAECs.

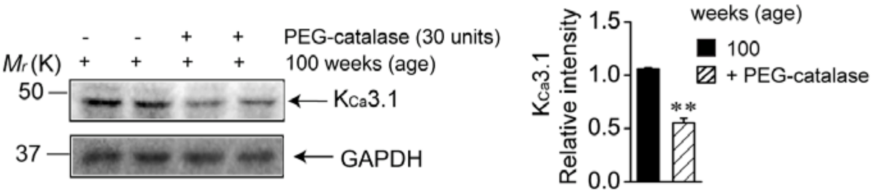

Fig. S5. **Changes in the levels of CerS and SLs in aorta or MAECs from CerS2 null mice.** **(A,B)** mRNA levels of CerS1-CerS6 **(A)** and levels of SLs **(B)** were measured in aortic tissues from 25-week-old CerS2 null and age-matched wild-type mice. Results were normalized to GAPDH levels (**A**). Aortic tissue from a mouse was used in each experiment, and bar graphs were made with pooled data from three experiments. **(C)** Levels of SLs were measured in cultured MAECs from 25-week-old CerS2 null and age-matched wild-type mice. Graphs were made with pooled data from four experiments performed with four different cultures. **P* < 0.05, ***P* < 0.01 versus wild-type.

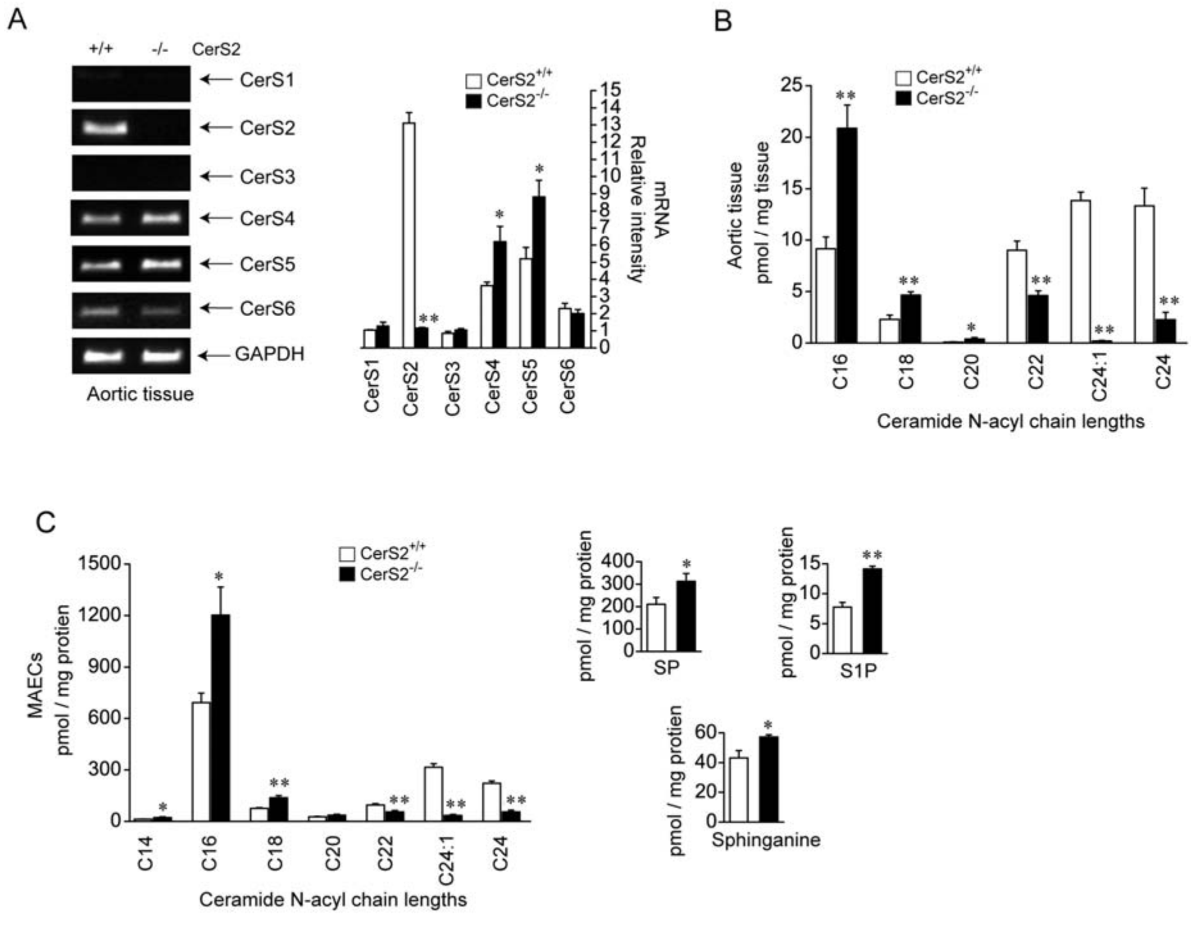

Fig. S6. Changes in expression levels of $K_{Ca}2.3$ in MAECs or aorta from CerS2 null, and aged wild-type mice.

(A) mRNA levels of $K_{Ca}2.3$ in MAECs or aorta from 25-week-old CerS2 null and age-matched wild-type mice. **(B,C)** mRNA (**B**) and protein (**C**) levels of $K_{Ca}2.3$ in aorta from aged (75-week-old) and young (15-week-old) wild-type mice. **(D)** Protein levels of $K_{Ca}2.3$ in MAECs from aged (100-week-old) and young (15-week-old) wild-type mice. Aortic tissue from a mouse was used in each experiment (**A-C**). Bar graphs were made with pooled data from three experiments performed with three different cultures or aortas. Results were normalized to GAPDH levels. $*P < 0.05$, $**P < 0.01$ versus age-matched wild-type.

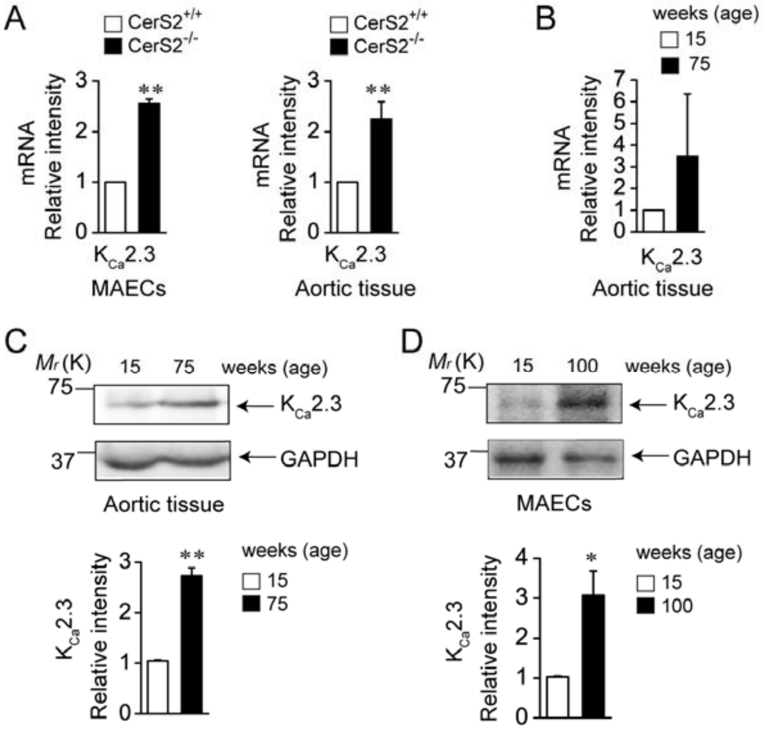

Supplement: Supplementary file 1 — Appendix S1 Supplementary materials and methods. Fig. S1 Changes in levels of TRXs in MAECs from CerS2 null mice. Fig. S2 Changes in levels of NO, ARG2 and NOX2 in MAECs from CerS2 null, catalase−/−/GPX−/−, and aged wild‐type mice. Fig. S3 l‐NOARG‐ and indomethacin‐resistant EDR by NS309. Fig. S4 PEG‐catalase reduced KCa3.1 levels in aged MAECs. Fig. S5 Changes in the levels of CerS and SLs in aorta or MAECs from CerS2 null mice. Fig. S6 Changes in expression levels of KCa2.3 in MAECs or aorta from CerS2 null, and aged wild‐type mice. [file ACEL-15-801-s001.pdf]
